# Efficacy and prognostic factors of first-line bevacizumab plus chemotherapy in elderly patients with advanced driver gene-negative lung adenocarcinoma: A retrospective study

**DOI:** 10.1097/MD.0000000000049939

**Published:** 2026-07-24

**Authors:** Shuiyao Li, Shaojun Wang, Ranhua Cao

**Affiliations:** a First Clinical Medical College, Inner Mongolia Medical University, Hohhot, China; b Department of Oncology, Affiliated Hospital of Inner Mongolia Medical University, Hohhot, China.

**Keywords:** bevacizumab, driver gene–negative, lung adenocarcinoma, prognostic model

## Abstract

We aimed to evaluate clinical outcomes and identify prognostic indicators in elderly patients with driver gene–negative advanced lung adenocarcinoma treated with first-line bevacizumab-based therapy. Elderly patients (≥65 years) with driver gene-negative advanced lung adenocarcinoma treated between January 2020 and December 2024 were retrospectively included. According to first-line treatment strategies, patients were divided into a chemotherapy-alone group and a bevacizumab plus chemotherapy group. Progression-free survival (PFS) and overall survival (OS) were analyzed using univariate and multivariate Cox regression models. A total of 169 patients were included. In the bevacizumab group, the median OS was 47 months and the median PFS was 24 months. Multivariate analysis showed that prognostic nutritional index (PNI), vascular endothelial growth factor A, carcinoembryonic antigen (CEA), carbohydrate antigen 125 (CA-125), and bevacizumab treatment were independent prognostic factors for PFS. Additionally, neutrophil-to-white blood cell ratio (NWR), neutrophil-to-lymphocyte ratio, PNI, vascular endothelial growth factor A, CA-125, and bevacizumab treatment were independently associated with OS. Our findings suggest that bevacizumab combined with chemotherapy is associated with favorable survival outcomes in elderly patients with driver gene-negative advanced lung adenocarcinoma. Inflammation-, nutrition-, and tumor burden-related biomarkers may help predict survival and assist in risk stratification.

## 1. Introduction

Lung cancer is one of the most common malignancies worldwide and remains the leading cause of cancer-related incidence and mortality in China.^[1]^ According to histological classification, lung cancer is broadly divided into small cell lung cancer and non-small cell lung cancer (NSCLC), of which NSCLC accounts for approximately 80 to 90% of cases, including lung adenocarcinoma and squamous cell carcinoma.^[2]^ Lung adenocarcinoma is the most prevalent subtype of NSCLC. Owing to its insidious clinical presentation, most patients are diagnosed at advanced stages, and the 5-year survival rate remains low at approximately 19.2%, indicating an overall poor prognosis.^[3]^ Advances in molecular biology have led to the identification of multiple oncogenic driver mutations, and targeted therapies have substantially improved survival outcomes in patients with advanced NSCLC harboring sensitive driver gene alterations.^[3,4]^ However, more than half of patients lack actionable driver mutations and therefore cannot benefit from targeted treatment, leaving systemic therapeutic options limited.^[3–5]^ Bevacizumab, an anti-angiogenic agent, has been recommended as part of systemic therapy for driver gene-negative advanced lung adenocarcinoma.^[6]^ Nevertheless, elderly patients frequently present with multiple comorbidities, age-related decline in organ function, and heterogeneous treatment tolerance, and not all patients derive equivalent survival benefit from bevacizumab-based therapy.^[7,8]^ Moreover, the potential for increased treatment-related adverse events and the associated economic burden may further restrict its clinical applicability in the absence of effective patient selection strategies.^[6–9]^ Identifying reliable prognostic and predictive indicators to stratify elderly patients who are most likely to benefit from bevacizumab treatment is therefore of substantial clinical importance.^[10]^ Although several prognostic models have been developed for risk assessment in NSCLC, evidence focusing on elderly patients, particularly those with lung adenocarcinoma receiving bevacizumab-based therapy, remains limited.^[10,11]^ Accordingly, this study retrospectively analyzed real-world clinical data to evaluate treatment outcomes and prognostic factors in elderly patients with driver gene-negative lung adenocarcinoma treated with bevacizumab, with the aim of developing a simple and practical prognostic assessment tool to support individualized clinical decision-making.

## 2. Methods

### 2.1. Patients

This was a retrospective study including 169 hospitalized elderly patients with driver gene–negative lung adenocarcinoma who received bevacizumab-based therapy at the Affiliated Hospital of Inner Mongolia Medical University between January 2020 and December 2024. All diagnoses were confirmed by histopathology or cytology. Follow-up was conducted until June 2025, with 166 patients successfully followed and 3 patients lost to follow-up, who were censored at the last observation. The cohort included 129 male and 40 female patients. Patients aged 65 to 70 years accounted for 43.20% (73/169), 71 to 80 years for 47.93% (81/169), and over 80 years for 8.88% (15/169). A history of smoking was present in 44.38% of patients, while 55.62% had no smoking history. Regarding disease stage, stage IV was predominant (71.60%, 121/169), and stage III accounted for 28.40% (48/169). T3/T4 tumors comprised 62.72% of cases, and N2/N3 lymph node involvement accounted for 56.21%. Tumor differentiation was mainly low (72.78%), followed by moderate (22.49%) and high (4.73%). Rates of distant metastases were 13.02% for brain, 10.65% for liver, 31.95% for lung, and 18.34% for bone. The study protocol was approved by the Institutional Review Board of the hospital (YKD202401116), and informed consent was obtained from all patients and their families. Inclusion criteria were as follows: histologically confirmed lung adenocarcinoma; molecular testing confirming driver gene–negative status, defined as the absence of pathogenic alterations in EGFR, KRAS, BRAF, HER2, ALK, ROS1, NTRK, RET, and MET, as determined by next-generation sequencing or real-time quantitative PCR (qPCR); unresectable stage III or IV according to tumor node metastasis classification; age ≥ 65 years, further classified as younger-old (<75 years) and older-old (≥75 years); at least one measurable lesion per Response Evaluation Criteria in Solid Tumorscriteria; first-line therapy with bevacizumab combined with chemotherapy for at least 4 cycles, with bevacizumab administered at 7.5 mg/kg intravenously every 21 days, with optional maintenance therapy; first-line chemotherapy with pemetrexed plus platinum, followed by optional pemetrexed maintenance; and complete clinical and follow-up data. Exclusion criteria included pathologically unconfirmed lung cancer, non-adenocarcinoma histology or driver gene–positive status, and incomplete efficacy or adverse event data. The treatment group received first-line bevacizumab plus chemotherapy, as described above. The control group received pemetrexed plus carboplatin for at least 4 cycles. Specifically, pemetrexed was administered at 500 mg/m^2^ intravenously on day 1, and carboplatin was administered intravenously on day 1 of each cycle, with the dose calculated using the Calvert formula (target AUC = 5). Treatment was repeated every 3 weeks. The platinum-based regimen was identical between the treatment and control groups. Survival time was calculated in months from the first day of chemotherapy to death or last follow-up. Patients who died from causes unrelated to lung cancer were censored. Follow-up was conducted via telephone or outpatient review, with the cutoff date of June 2025.

### 2.2. Study methods

Peripheral venous blood samples were obtained from all patients within one week prior to the initiation of first-line treatment as part of routine clinical evaluation. Laboratory and immunological parameters were retrospectively collected from the hospital’s electronic medical record system. Serum tumor markers, including carcinoembryonic antigen (CEA) and carbohydrate antigen 125 (CA-125), were measured using standard chemiluminescence immunoassays in the central laboratory of the Affiliated Hospital of Inner Mongolia Medical University. Nutritional and immune status were evaluated using the prognostic nutritional index (PNI), calculated as serum albumin (g/L) + 5 × lymphocyte count (10^9^/L). Angiogenesis-related indicators, including vascular endothelial growth factor A (VEGF-A) levels, were also assessed. Systemic inflammatory markers were calculated based on complete blood count results, including neutrophil-to-white blood cell ratio (NWR), monocyte-to-white blood cell ratio (MWR), lymphocyte-to-white blood cell ratio (LWR),platelet-to-white blood cell ratio (PWR), neutrophil-to-lymphocyte ratio (NLR), monocyte-to-lymphocyte ratio (MLR), and platelet-to-lymphocyte ratio (PLR). Peripheral blood lymphocyte subsets, including CD4^+^ T cells and CD8^+^ T cells, were measured as part of routine clinical immune function testing using flow cytometry in the hospital’s clinical laboratory. The CD4^+^/CD8^+^ T cell ratio was calculated accordingly. All immune cell data were retrospectively retrieved from medical records. Long-term treatment efficacy was evaluated using progression-free survival (PFS), defined as the time from initiation of therapy to disease progression or death, and overall survival (OS), defined as the time from initiation of therapy to death from any cause. Patients without events at the end of follow-up were treated as censored data in the analysis.

### 2.3. Statistical analysis

Data were analyzed using SPSS version 27.0. Continuous variables were expressed as mean ± SD (normal distribution) or median (IQR) (non-normal distribution). Categorical data were presented as numbers and percentages. Group differences were compared using Student *t* test or Mann–Whitney *U* test for continuous variables, and the Chi-square test for categorical variables. Kaplan–Meier survival analysis was performed for univariate analysis, and survival curves were compared between groups using the log-rank test. Variables with statistical significance in univariate analysis were further included in a multivariate Cox proportional hazards regression model using a stepwise backward variable selection method.^[12]^ A two-sided *P* value < .05 was considered statistically significant.

## 3. Results

### 3.1. Baseline characteristics of the study population

A total of 169 patients with advanced lung adenocarcinoma were included in this study, with a predominance of male patients (76.33%). In the chemotherapy-alone group, males accounted for 80.95%, while in the combination therapy group, males accounted for 71.76%. Regarding age distribution, patients aged 65 to 70 years and 71 to 80 years comprised the majority of the cohort. The 2 groups were well-balanced with respect to general clinical characteristics, including sex, age, smoking history, disease stage, tumor differentiation, and metastasis-related features, with no statistically significant differences observed between groups (all *P* > .05). Baseline characteristics were therefore comparable between the 2 treatment groups (detailed in Table 1).

**Table 1:** Baseline clinical characteristics of patients with advanced lung adenocarcinoma.

Clinical characteristics	Total (n = 169)	Chemotherapy alone (n = 84)	Bevacizumab + chemotherapy (n = 85)	χ^2^		*P*
Sex						
Male	129 (76.33%)	68 (80.95%)	61 (71.76%)	1.974	.160	
Female	40 (23.67%)	16 (19.05%)	24 (28.24%)			
Age (yr)						
65–70	73 (43.20%)	38 (45.24%)	35 (41.18%)	0.493	.782	
71–80	81 (47.93%)	38 (45.24%)	43 (50.59%)			
>80	15 (8.88%)	8 (9.52%)	7 (8.24%)			
Smoking history						
No	94 (55.62%)	50 (59.52%)	44 (51.76%)	1.03	.310	
Yes	75 (44.38%)	34 (40.48%)	41 (48.24%)			
Stage						
III	48 (28.40%)	29 (34.52%)	19 (22.35%)	3.078	.079	
IV	121 (71.60%)	55 (65.48%)	66 (77.65%)			
T stage						
T1/T2	63 (37.28%)	36 (42.86%)	27 (31.76%)	2.223	.136	
T3/T4	106 (62.72%)	48 (57.14%)	58 (68.24%)			
N stage						
N0/N1	74 (43.79%)	41 (48.81%)	33 (38.82%)	1.712	.191	
N2/N3	95 (56.21%)	43 (51.19%)	52 (61.18%)			
Tumor differentiation						
Poor	123 (72.78%)	60 (71.43%)	63 (74.12%)	0.567	.753	
Moderate	38 (22.49%)	19 (22.62%)	19 (22.35%)			
Well	8 (4.73%)	5 (5.95%)	3 (3.53%)			
Brain metastasis						
No	147 (86.98%)	75 (89.29%)	72 (84.71%)	0.783	.376	
Yes	22 (13.02%)	9 (10.71%)	13 (15.29%)			
Liver metastasis						
No	151 (89.35%)	74 (88.10%)	77 (90.59%)	0.276	.599	
Yes	18 (10.65%)	10 (11.90%)	8 (9.41%)			
Lung metastasis						
No	115 (68.05%)	61 (72.62%)	54 (63.53%)	1.605	.205	
Yes	54 (31.95%)	23 (27.38%)	31 (36.47%)			
Bone metastasis						
No	138 (81.66%)	67 (79.76%)	71 (83.53%)	0.400	.527	
Yes	31 (18.34%)	17 (20.24%)	14 (16.47%)			

TNM = tumor node metastasis, χ^2^ = Chi-Square statistic.

### 3.2. Comparison of pretreatment inflammation-related indicators between the two groups

We compared 7 inflammation-related indicators (NWR, MWR, LWR, PWR, NLR, MLR, and PLR) between the chemotherapy-alone and bevacizumab-plus-chemotherapy groups. The results demonstrated that patients receiving bevacizumab plus chemotherapy showed significantly lower levels of NWR, MWR, PWR, and MLR, along with a higher level of LWR, compared with those receiving chemotherapy alone. These findings suggest that bevacizumab-based combination therapy may be associated with a more favorable inflammatory status. In contrast, no statistically significant differences were observed in NLR and PLR between the 2 groups. Detailed results are presented in Table 2.

**Table 2:** Comparison of inflammation-related indicators between chemotherapy and bevacizumab plus chemotherapy groups [median (IQR)].

Group	n	NWR	MWR	LWR	PWR	NLR	MLR	PLR
Chemotherapy	84	0.68 (0.58, 0.78)	0.08 (0.06, 0.10)	0.25 (0.16, 0.33)	38.46 (28.73, 45.74)	3.04 (1.84, 4.73)	0.36 (0.25, 0.50)	144.04 (110.60, 219.48)
Bevacizumab + Chemotherapy	85	0.65 (0.55, 0.70)	0.07 (0.06, 0.09)	0.27 (0.22, 0.33)	35.00 (27.64, 39.90)	2.44 (1.84, 3.86)	0.28 (0.20, 0.36)	137.65 (103.35, 198.81)
*Z*		−2.140	−3.046	−1.990	−1.968	−1.041	−2.976	−1.083
*P*		.032	.002	.047	.049	.298	.003	.279

All variables in Table 2 were non-normally distributed and are expressed as median (interquartile range, IQR). *Z* and *P* values were calculated using the Mann–Whitney *U* test.

LWR = lymphocyte to white blood cell ratio, MLR = monocyte to lymphocyte ratio, MWR = monocyte to white blood cell ratio, NLR = neutrophil to lymphocyte ratio, NWR = neutrophil to white blood cell ratio, PLR = platelet to lymphocyte ratio, PWR = platelet to white blood cell ratio.

### 3.3. Comparison of pretreatment prognostic nutritional index (PNI) between the two groups

We analyzed the PNI, VEGF-A, and CD4^+^/CD8^+^ ratio in the chemotherapy-alone and bevacizumab-plus-chemotherapy groups. The results demonstrated that patients in the combination group exhibited a significantly higher PNI and CD4^+^/CD8^+^ ratio, along with a markedly lower level of VEGF-A, compared with those receiving chemotherapy alone. These findings suggest that bevacizumab-based combination therapy may be associated with improved nutritional status, enhanced immune balance, and effective inhibition of angiogenesis. Detailed results are presented in Table 3.

**Table 3:** Comparison of PNI, VEGF-A, and CD4^+^/CD8^+^ ratio between chemotherapy and bevacizumab plus chemotherapy groups.

Group	n	PNI [median (IQR)]	VEGF-A (pg/mL) [mean ± SD]	CD4 (cells/µL) [median (IQR)]	CD8 (cells/µL) [median (IQR)]	CD4^+^/CD8^+^ [median (IQR)]
Chemotherapy	84	45.75 (42.74, 48.80)	95.89 ± 24.84	504.50 (373.50, 748.75)	718.50 (595.50, 833.75)	0.76 (0.59, 1.03)
Bevacizumab + chemotherapy	85	47.10 (44.33, 51.80)	79.22 ± 32.14	583.00 (434.50, 818.50)	701.00 (517.50, 796.00)	0.93 (0.74, 1.18)
*Z*/*t*		−2.443	3.769	−1.317	−1.589	−3.083
*P*		.015	.000	.188	.112	.002

Continuous variables are expressed as mean ± standard deviation or median (interquartile range, IQR) according to data distribution. VEGF-A was normally distributed and analyzed using the independent samples *t*-test, while PNI, CD4^+^, CD8^+^, and CD4^+^/CD8^+^ ratio were non-normally distributed and analyzed using the Mann–Whitney *U* test.

PNI = prognostic nutritional index, VEGF-A = vascular endothelial growth factor A.

### 3.4. Comparison of pretreatment tumor markers between the two groups

We compared serum levels of CEA and CA-125 between the chemotherapy-alone and bevacizumab-plus-chemotherapy groups. Patients receiving combination therapy exhibited significantly lower levels of both CEA and CA-125 compared with those treated with chemotherapy alone, indicating a greater reduction in tumor-associated markers and tumor burden with bevacizumab-based therapy. These findings are summarized in Table 4.

**Table 4:** Comparison of CEA and CA-125 levels between chemotherapy and bevacizumab plus chemotherapy groups.

Group	n	CEA (ng/mL) [Median (IQR)]	CA-125 (U/mL) [Mean ± SD]
Chemotherapy	84	46.16 (34.59, 57.29)	67.81 ± 18.23
Bevacizumab + Chemotherapy	85	39.54 (22.58, 53.64)	50.46 ± 19.53
*Z*/*t*		−2.283	5.970
*P*		.022	.000

Continuous variables are expressed as mean ± standard deviation or median (interquartile range, IQR) according to data distribution.CA-125 was normally distributed and analyzed using the independent samples *t*-test, while CEA was non-normally distributed and analyzed using the Mann–Whitney *U* test.

CEA = carcinoembryonic antigen, CA-125 = carbohydrate antigen 125.

### 3.5. Analysis of progression-free survival (PFS) and overall survival between the two groups

**Figure 1.**
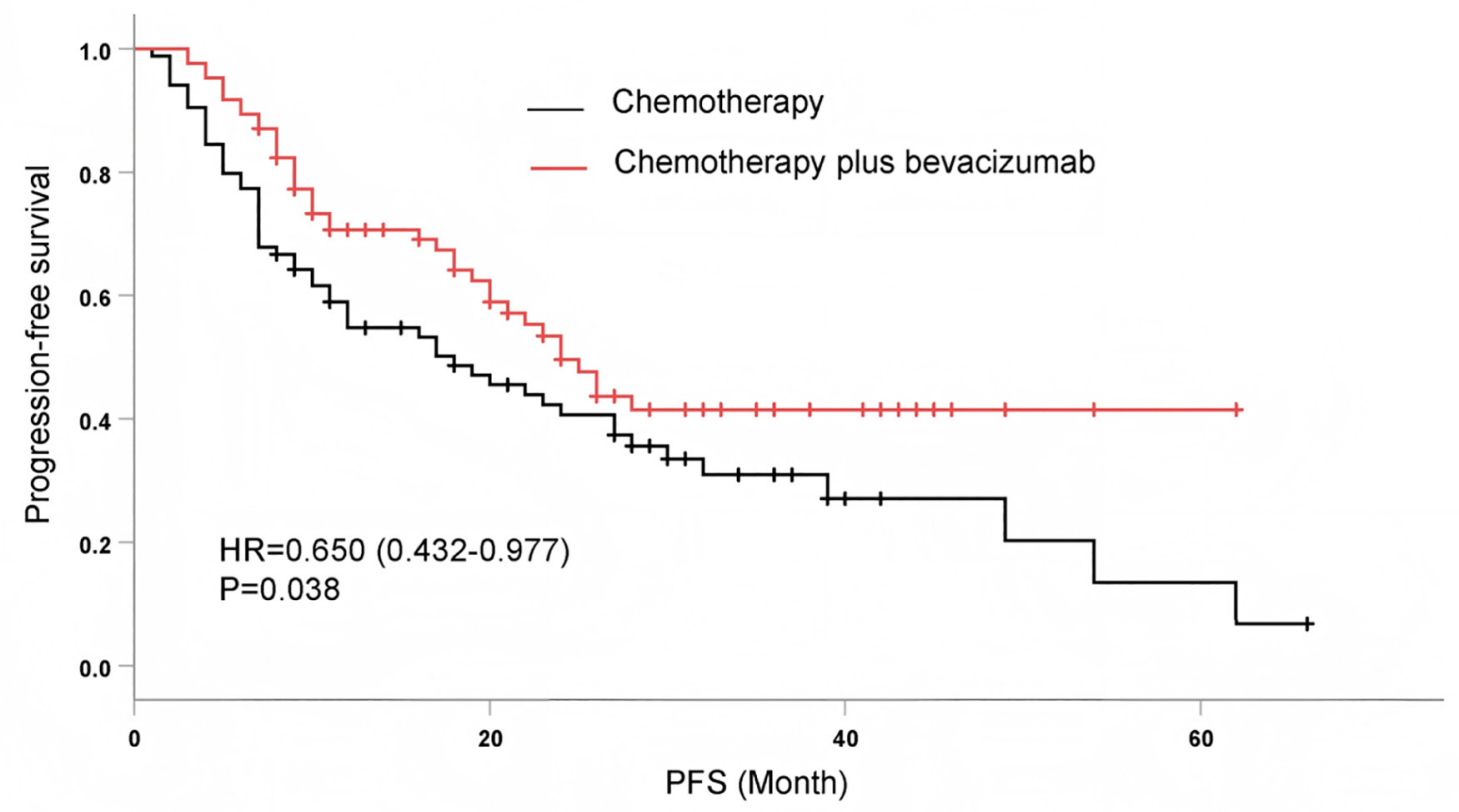
Comparison of progression-free survival between the chemotherapy and bevacizumab plus chemotherapy groups.

**Figure 2.**
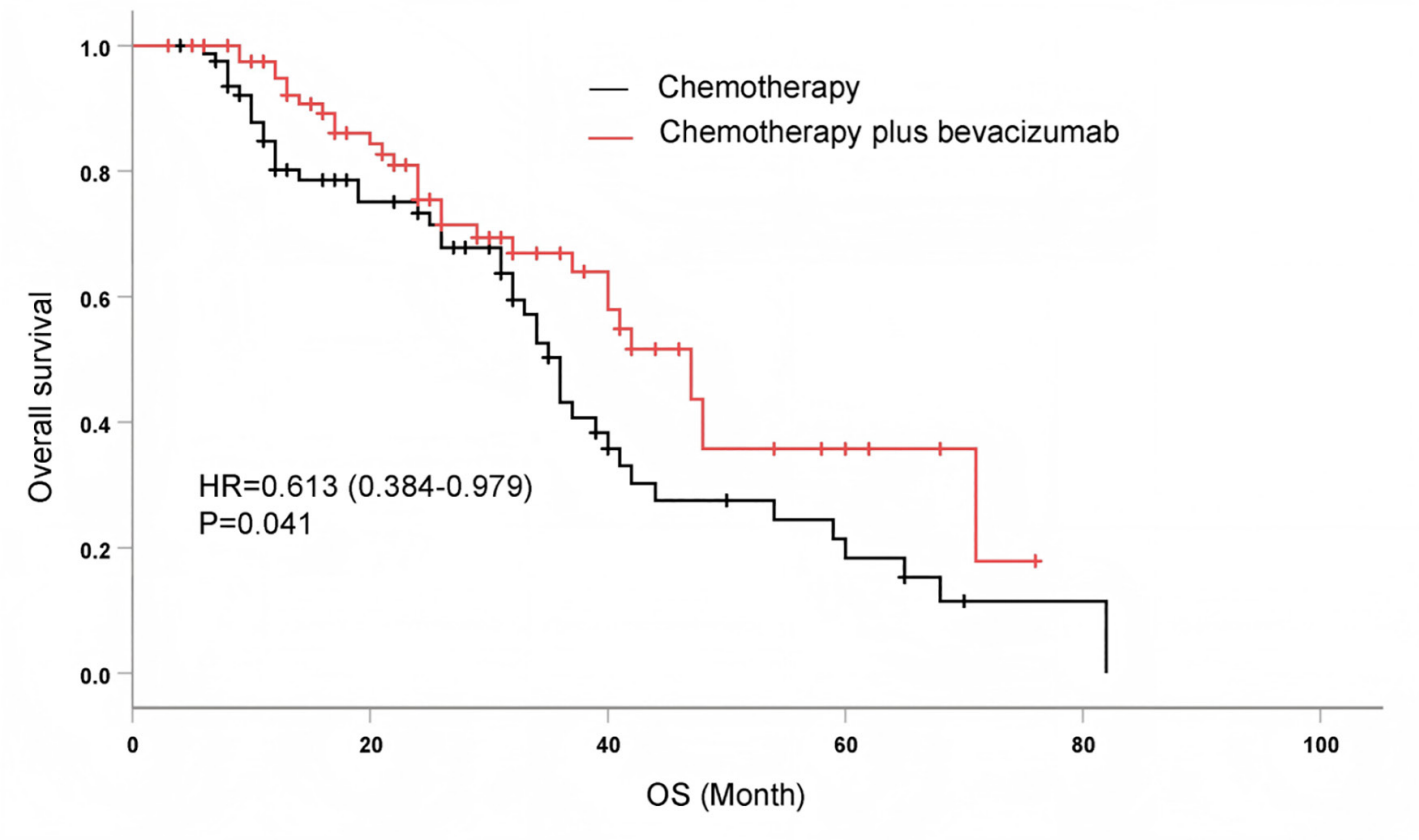
Comparison of overall survival between the chemotherapy and bevacizumab plus chemotherapy groups.

To assess PFS differences between the chemotherapy-alone and bevacizumab-plus-chemotherapy groups, Kaplan–Meier survival curves were plotted and compared using the Log-rank test, with median survival time and hazard ratio (HR) analyzed. The Log-rank test indicated a significant difference in PFS between the 2 groups (χ^2^ = 4.485, *P* = .038). Median PFS was 18.0 months in the chemotherapy-alone group and 24.0 months in the combination group, with longer PFS observed in the bevacizumab-plus-chemotherapy group. The HR for the combination group relative to the chemotherapy-alone group was 0.650 (95% confidence interval (CI): 0.432–0.977), indicating that patients in the bevacizumab-plus-chemotherapy group had significantly improved PFS compared with those in the chemotherapy-alone group (Fig. 1). To evaluate OS differences between the 2 groups, Kaplan–Meier curves were plotted and compared using the Log-rank test, with median survival time and HR analyzed. The Log-rank test indicated a significant difference in OS between the 2 groups (χ^2^ = 4.364, *P* = .041). Median OS was 36.0 months in the chemotherapy-alone group and 47.0 months in the combination group, with longer OS observed in the combination group. The HR for the combination group relative to the chemotherapy-alone group was 0.613 (95% CI: 0.384–0.979), suggesting that patients in the combination group had significantly improved survival compared with the chemotherapy-alone group (Fig. 2).

### 3.6. Analysis of prognostic factors

To evaluate the impact of clinical and laboratory indicators on PFS, univariate and multivariate Cox proportional hazards analyses were performed. Univariate analysis identified NWR, PNI, VEGF-A, CEA, CA-125, and bevacizumab treatment as significant factors (*P* < .05). Elevated NWR, VEGF-A, CEA, and CA-125 were associated with shorter PFS, while higher PNI and bevacizumab treatment were protective. Variables with *P* < .1 were included in multivariate analysis. Results showed that PNI, VEGF-A, CEA, CA-125, and bevacizumab remained independent predictors of PFS (*P* < .05). Specifically, higher PNI and bevacizumab treatment significantly improved PFS, whereas elevated VEGF-A, CEA, and CA-125 remained independent risk factors. Other factors did not significantly affect PFS (*P* > .05) (Table 5). These findings suggest that nutritional, tumor, and angiogenesis markers, together with bevacizumab therapy, can stratify patients according to their expected PFS. To evaluate the impact of clinical and laboratory indicators on OS, univariate and multivariate Cox proportional hazards analyses were performed. Univariate analysis identified NWR, NLR, PLR, PNI, VEGF-A, CEA, CA-125, and bevacizumab treatment as significant factors (*P* < .05). Elevated NWR, NLR, PLR, VEGF-A, CEA, and CA-125 were associated with worse OS, whereas higher PNI and bevacizumab therapy were protective. Variables with *P* < .1 were included in multivariate analysis. The results showed that NWR, NLR, PNI, VEGF-A, CA-125, and bevacizumab remained independent predictors of OS (*P* < .05). Specifically, higher PNI and bevacizumab treatment significantly improved OS, whereas elevated NWR, NLR, VEGF-A, and CA-125 remained independent risk factors. Other variables did not significantly affect OS (*P* > .05) (Table 6). These findings suggest that systemic inflammation, nutritional status, angiogenesis markers, and bevacizumab therapy can stratify patients according to expected OS.

**Table 5:** Univariate and multivariate Cox regression analysis of factors for progression-free survival.

Variable	Univariate Cox			Multivariate Cox		
	HR (95% CI)	Wald	*P*	HR (95% CI)	Wald	*P*
NWR	2.478 (1.242, 4.942)	6.636	.010	1.005 (0.426, 2.371)	0.000	.991
MWR	6.031 (0.025, 1480.480)	0.410	.522	–	–	–
LWR	0.715 (0.137, 3.727)	0.158	.691	–	–	–
PWR	1.015 (0.996, 1.035)	2.542	.111	–	–	–
NLR	0.911 (0.829, 1.001)	3.731	.053	0.920 (0.833, 1.017)	2.637	.104
MLR	0.706 (0.327, 1.522)	0.790	.374	-	–	–
PLR	0.999 (0.997, 1.001)	0.666	.414	–	–	–
PNI	0.951 (0.912, 0.991)	5.686	.017	0.943 (0.919, 0.986)	4.923	.026
VEGF-A	1.009 (1.002, 1.016)	5.738	.017	1.014 (1.006, 1.193)	6.087	.014
CD4^+^/CD8^+^	1.453 (0.701, 3.011)	1.011	.315	–	–	–
CEA (g/mL)	1.019 (1.009, 1.029)	13.304	.000	1.014 (1.003, 1.025)	5.873	.015
CA-125 (U/mL)	1.026 (1.017, 1.036)	28.467	.000	1.025 (1.012, 1.037)	15.783	.000
Bevacizumab	0.650 (0.432, 0.977)	4.283	.038	0.593 (0.414, 0.826)	7.626	.006

CA-125 = carbohydrate antigen 125, CEA = carcinoembryonic antigen, CI = confidence interval, HR = hazard ratio, LWR = lymphocyte to white blood cell ratio, MLR = monocyte to lymphocyte ratio, MWR = monocyte to white blood cell ratio, NLR = neutrophil to lymphocyte ratio, NSCLC = non-small cell lung cancer, NWR = neutrophil to white blood cell ratio, PLR = platelet to lymphocyte ratio, PNI = prognostic nutritional index, PWR = platelet to white blood cell ratio, RECIST = response evaluation criteria in solid tumors, SCLC = small cell lung cancer, TNM = tumor node metastasis, VEGF-A = vascular endothelial growth factor A.

**Table 6:** Univariate and multivariate Cox regression analysis of factors for overall survival.

Variable	Univariate Cox			Multivariate Cox		
	HR (95% CI)	Wald	*P*	HR (95% CI)	Wald	*P*
NWR	2.181 (1.043, 4.559)	4.297	.038	1.757 (1.367, 4.197)	4.848	.028
MWR	7.056 (0.043, 68.433)	0.862	.353	–	–	–
LWR	0.850 (0.146, 4.964)	0.033	.857	–	–	–
PWR	1.011 (0.990, 1.033)	1.057	.304	–	–	–
NLR	1.357 (1.063, 1.961)	6.905	.009	1.483 (1.195, 2.322)	3.944	.047
MLR	0.510 (0.212, 1.223)	2.280	.131	–	–	–
PLR	1.127 (1.043, 1.681)	4.053	.044	1.303 (0.995, 2.003)	0.346	.556
PNI	0.918 (0.872, 0.968)	10.218	.001	0.911 (0.861, 0.965)	10.290	.001
VEGF-A	1.013 (1.005, 1.022)	9.461	.002	1.013 (1.004, 1.022)	7.412	.006
CD4^+^/CD8^+^	1.225 (0.549, 2.734)	0.246	.620	–	–	–
CEA (g/mL)	1.016 (1.005, 1.028)	7.828	.005	1.010 (0.996, 1.024)	1.920	.166
CA-125 (U/mL)	1.012 (1.001, 1.024)	4.761	.029	1.218 (1.098, 1.643)	4.315	.038
Bevacizumab	0.613 (0.384, 0.979)	4.190	.041	0.831 (0.479, 0.977)	4.434	.035

CA-125 = carbohydrate antigen 125, CEA = carcinoembryonic antigen, CI = confidence interval, HR = hazard ratio, LWR = lymphocyte-to-white blood cell ratio, MLR = monocyte-to-lymphocyte ratio, MWR = monocyte-to-white blood cell ratio, NLR = neutrophil-to-lymphocyte ratio, NSCLC = non-small cell lung cancer, NWR = neutrophil-to-white blood cell ratio, PLR = platelet-to-lymphocyte ratio, PNI = prognostic nutritional index, PWR = platelet-to-white blood cell ratio, RECIST = response evaluation criteria in solid tumors, SCLC = small cell lung cancer, TNM = tumor node metastasis, VEGF-A = vascular endothelial growth factor A.

## 4. Discussion

With population aging, elderly patients (≥70 years) with advanced NSCLC are becoming increasingly prevalent in clinical practice, posing unique challenges for treatment selection.^[3,4,7]^ Previous studies have identified male sex, poor performance status, multiple distant metastases, and recent weight loss as negative prognostic factors for OS in elderly patients.^[13]^ However, prognostic stratification for specific therapeutic regimens, such as bevacizumab combined with chemotherapy, remains limited.In this study, we retrospectively analyzed 169 elderly patients with advanced lung adenocarcinoma to systematically evaluate the real-world efficacy of bevacizumab combined with chemotherapy and to identify independent prognostic factors for PFS and OS. The results demonstrated that bevacizumab combination therapy significantly improved both PFS and OS, with median PFS and OS of 24 and 47 months, respectively, outperforming chemotherapy alone. Multivariate Cox analysis revealed that PNI, VEGF-A, CEA, CA-125, and bevacizumab were independent predictors of PFS, whereas NWR, NLR, PNI, VEGF-A, CA-125, and bevacizumab independently predicted OS.

Tumor progression and its microenvironment can induce systemic chronic inflammation, which remodels the extracellular matrix and promotes angiogenesis and lymphangiogenesis, thereby facilitating tumor growth and metastasis.^[14,15]^ The significant associations of NWR, NLR, and PNI in our cohort suggest that systemic inflammation and the nutritional-immune microenvironment are key biological determinants of therapeutic response and long-term survival in elderly patients, beyond traditional tumor burden indicators. Previously published evidence has demonstrated that elevated NWR and NLR, which reflect heightened systemic inflammatory responses, have been associated with poorer prognosis. This association may be explained by neutrophil-derived pro-inflammatory cytokines (e.g., tumor necrosis factor alpha) and pro-angiogenic factors (e.g., VEGF), which may promote tumor progression and suppress lymphocyte-mediated antitumor responses.^[16–19]^ CD4^+^ and CD8^+^ T lymphocyte counts were collected in our cohort, and higher CD4^+^/CD8^+^ ratios were observed in patients with better prognosis, suggesting that preserved T cell-mediated immunity contributes to improved outcomes. Cytotoxic CD8^+^ T cells primarily mediate antitumor effects through the perforin/ granzyme pathway, underscoring the importance of direct cytolytic activity in controlling tumor progression.^[20–22]^ Previous literature has also established systemic inflammatory markers as robust prognostic indicators in multiple cancers, consistent with our findings.^[23–25]^

Nutrition plays a critical modulatory role at the intersection of inflammation and immunity.^[26–28]^ Serum albumin, a core nutritional marker, is negatively regulated by inflammatory cytokines, and malnutrition often coexists with immune deficiency.^[29]^ The PNI integrates both aspects.^[30]^ Our analysis confirmed that higher baseline PNI independently predicted longer PFS and OS, suggesting that maintaining or improving PNI through nutritional interventions may enhance immune function, treatment tolerance, and ultimately prognosis in elderly, driver-gene-negative lung adenocarcinoma patients.

VEGF, a mitogen specific to endothelial cells, is a key regulator of angiogenesis.^[31,32]^ Angiogenesis critically impacts patient survival, and numerous studies have demonstrated VEGF’s prognostic role in NSCLC.^[32–34]^ In this study, VEGF-A, the target of bevacizumab, was significantly associated with poorer PFS and OS, supporting the biological rationale for anti-angiogenic therapy in patients with high VEGF expression. Notably, bevacizumab itself remained an independent protective factor for PFS and OS, indicating additional mechanisms, such as tumor vessel normalization and improved chemotherapy delivery, may contribute to its antitumor effect.

Serum tumor markers, including CEA and CA-125, are widely used for prognosis and treatment monitoring.^[35,36]^ Consistent with previous reports, these markers showed strong predictive ability in our cohort, maintaining significance in multivariate models, highlighting the continued relevance of tumor burden and biological behavior in elderly patients.

In summary, this study proposes a simple, elderly-specific prognostic model for advanced lung adenocarcinoma. Unlike existing models derived from all-age cohorts, our model is fully developed in an elderly population, addressing limitations of prior tools. Limitations include the single-center, retrospective design, relatively small sample size, particularly for patients > 80 years, absence of emerging biomarkers such as programmed death ligand 1 or tumor mutational burden, and lack of comprehensive inflammatory markers (e.g., IL-6) that may further refine risk stratification. While internal validation suggests reliability, external validation is necessary to confirm its clinical applicability.

Overall, this study provides the first comprehensive evaluation of first-line bevacizumab plus chemotherapy in elderly patients with advanced lung adenocarcinoma. PNI, VEGF-A, CEA, CA-125, systemic inflammatory indices (NWR, NLR), and bevacizumab itself serve as simple, effective prognostic tools to identify patients likely to benefit and guide individualized treatment strategies. Prospective studies integrating multidimensional biomarkers are warranted to further refine prognostic modeling and optimize therapeutic decision-making for elderly lung cancer patients.

## Author Contributions

**Investigation:** Shuiyao Li.

**Methodology:** Shaojun Wang.

**Writing – original draft:** Shuiyao Li, Ranhua Cao.

**Writing – review & editing:** Ranhua Cao.
